# An interview with Arno Locks

**DOI:** 10.1590/2176-9451.19.5.031-044.int

**Published:** 2014

**Authors:** Carla D’Agostini Derech, Gerson Luiz Ulema Ribeiro, José Nelson Mucha, Luiz Gonzaga Gandini Jr, Maurício Tatsuei Sakima

**Affiliations:** Professor, Postgraduate program in Orthodontics, Federal University of Santa Catarina (UFSC). MSc in Orthodontics, Federal University of Rio de Janeiro (UFRJ). PhD in Orthodontics, Federal University of Rio de Janeiro (UFRJ). Chairperson, Brazilian Association of Orthodontics and Facial Orthopedics (ABOR / Santa Catarina).; Adjunct professor, Department of Orthodontics, Federal University of Santa Catarina (UFSC). Postdoc in Orthodontics, Baylor College of Dentistry, USA. PhD in Orthodontics, Federal University of Rio de Janeiro (UFRJ). MSc in Orthodontics, Federal University of Rio de Janeiro (UFRJ).; Full professor, Department of Orthodontics, Fluminense Federal University (UFF). PhD in Orthodontics, Federal University of Rio de Janeiro (UFRJ). MSc in Orthodontics, Federal University of Rio de Janeiro (UFRJ).; Full professor, State University of São Paulo (UNESP). Postdoc in Orthodontics, Baylor College of Dentistry, USA. PhD in Orthodontics, State University of São Paulo (UNESP) / Araraquara. MSc in Orthodontics, State University of São Paulo (UNESP) / Araraquara. Specialist in Orthodontics, Association of Dental Surgeons / Araraquara.; Assistant professor and PhD, State University of São Paulo (UNESP) / Araraquara. Postdoc, University of Aarhus Dental College, Denmark. PhD in Orthodontics, State University of São Paulo (UNESP). MSc in Orthodontics, State University of São Paulo (UNESP).

**Figure:**
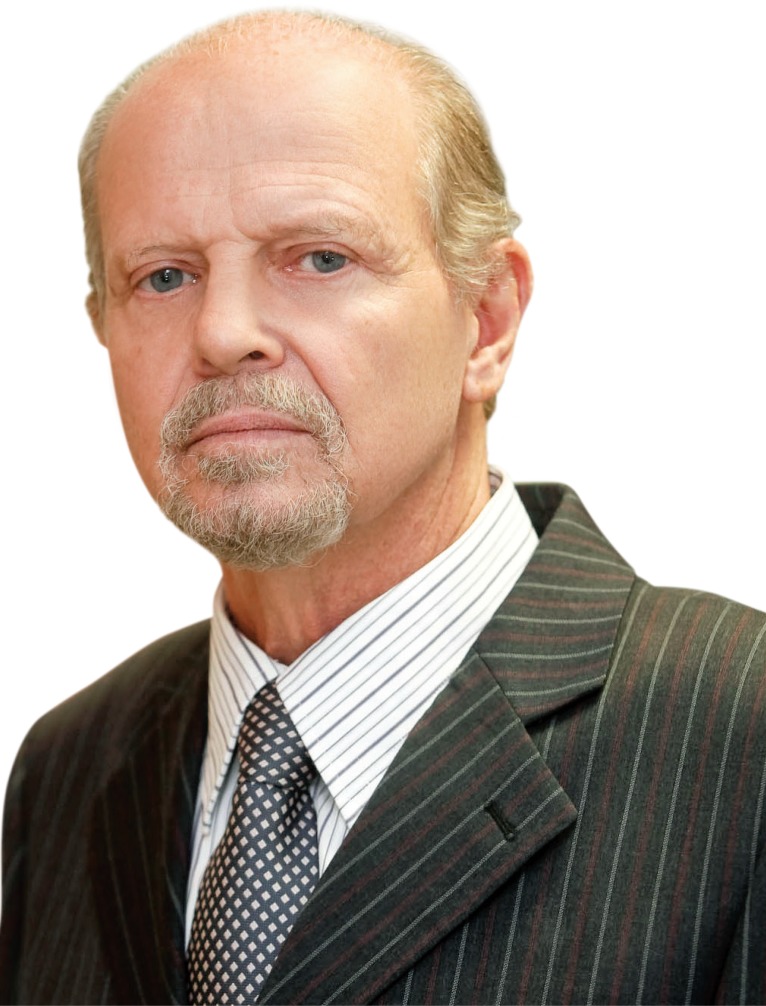

I met Arno Locks in the 80s when he was my undergraduate professor. However, he will always be my professor; not only in Orthodontics, but also in ethics and fight for the causes of Dentistry. He loves what he does. And those who love, also care, listen and get involved. As undergraduate students, we were aware that a forward-thinking professional who devoted special attention to his students was right in front of us. As his colleague, I fondly remember when he invited me to join the orthodontic postgraduate program at the Federal University of Santa Catarina (UFSC), always encouraging me to express my opinions in a critical and constructive manner. He is always open to discussion! I found these moments in which we are able to express our admiration for a colleague as Prof. Arno very especial, and I have fostered this opportunity with great pleasure. He was born in Braço do Norte, a town located in the Brazilian state of Santa Catarina. As a little child, his family moved to the metropolitan area of Florianópolis, in Biguaçu, where he began his career. After receiving his DDS degree from UFSC in 1973 and setting his own office, Prof. Arno Locks moved to Rio de Janeiro where he entered the postgraduate program in Orthodontics. A few years later, he moved back to Florianópolis as a Master in Orthodontics and professor at the Federal University of Santa Catarina (UFSC). As a renowned professor with his own office successfully set in Florianópolis, he went on with his career and entered the PhD program at State University of São Paulo (UNESP) in the city of Araraquara. That was when he got to know the segmented arch technique and mechanics with predetermined force system. In 2004, he faced a new challenge. However, for Prof. Arno, challenge is fuel and he entered the Postdoctoral program at Aarhus University (Denmark) under supervision of Prof Birte Melsen. I am privileged to have a close relationship with such a generous person who is continuously motivated and demonstrates, at his office or university, extensive experience as well as ability to listen and enjoy teaching and learning. His attitude explains his extensive knowledge which he generously shares as a clinician and researcher. At the same time, his political posture toward an ethic, scientific-based Orthodontics has always been present in his fight for professional alliance. Brazilian Orthodontics owes great respect to Prof. Arno Locks. We are deeply grateful for the assemblies and meetings during which low-quality mercantilist Orthodontics tried to advance, but came across the strong and decisive voice of someone who is strongly committed to his ideals with courage and love. Thank you very much! 

Carla D'Agostini Derech

What changes were brought about to your orthodontic practice after receiving PhD (State University of São Paulo - UNESP) and Postdoctoral (Aarhus University) degrees from two institutions that have been eagerly teaching orthodontic treatment conducted by means of segmented system of forces and arch mechanics? Luiz Gonzaga Gandini Jr.

## In which respect of biomechanics do you highlight Prof. Birte Melsen? Maurício Tatsuei Sakima

Initially, I would like to thank Prof. Birte Melsen profusely for granting me the privilege of conducting my postdoctoral research at Aarhus University where I acquired unimaginable scientific knowledge on biomechanics.

Prof. Birte Melsen, with great wisdom and creativity, considerably improved the segmented arch technique. She has conducted numerous researches throughout her academic career and has taught us that biomechanics as a science does not consist in installing orthodontic appliances, only. Regardless of prescription, biomechanics is more than tying archwire and waiting for everything to be solved as if by magic without even a basic knowledge of what is going on.

Prof. Birte Melsen has demonstrated that biomechanics consists in using proper system of forces individualized to address the needs of each case.

As she has repeatedly mentioned, the clinician should plan biomechanics before treatment onset, determining which teeth will be moved in which direction of the three planes of space; in addition to treatment goals. If we do not know where we are going, it is impossible to get there.^1^

Likewise, I have a lot to thank to the Department of Orthodontics at the State University of São Paulo (UNESP) in Araraquara where I was welcomed in the 90's for my PhD. During three years, I had to travel extensively; however, with great joy and pleasure, as I was fulfilling my desires and meeting good friends.

My PhD and Postdoctoral degrees provided me with important and indispensable academic/professional growth, since the knowledge I acquired about the segmented arch technique allowed me to treat more complex cases with which I used to have great difficulty. 

Some cases can only be treated by means of the segmented arch technique; otherwise, there will be no efficient outcomes. More severe cases in need of individualized systems of forces, as it is the case of movement of periodontally compromised teeth, cannot be treated differently.

**Figure 1:**
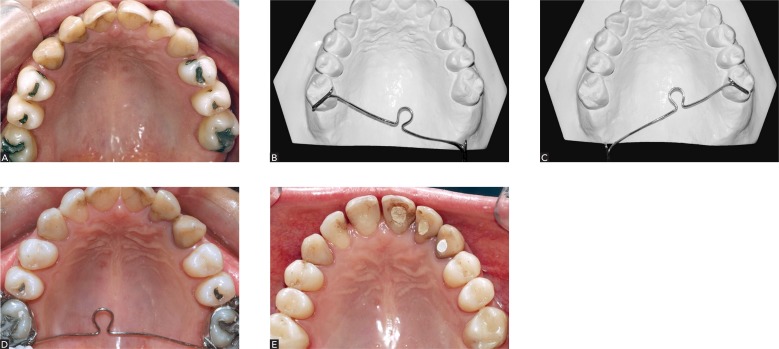
A) Clinical case revealing totally tipped molars. B, C) Dental casts revealing activation of transpalatal arch in VI geometry. D) Activated and properly installed transpalatal arch. F) Final outcomes of molar correction.

**Figure 2:**
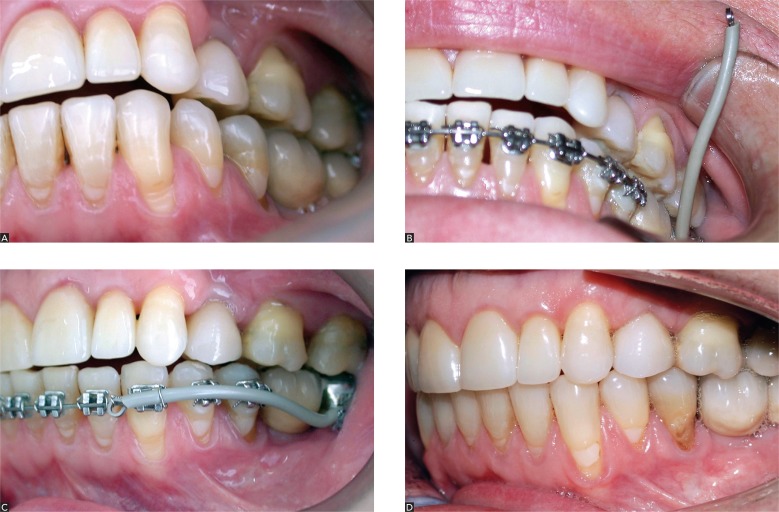
Clinical case using cantilever and implant anchorage with Straight-Wire brackets to correct open bite and asymmetry to the left.

The segmented arch technique with devices such as transpalatal arch, lingual arch and cantilevers allows us to obtain measurable controlled forces and predictable movements. These devices favor a much more efficient biomechanics (Figs 1 and 2).

## Still with regard to biomechanics, what remains the same since you graduated from UFRJ in 1979? José Nelson Mucha

The essentials acquired during my Master's course at UFRJ remain exactly the same. And I keep them alive with pride and affection.

At that distinguished institution I learned how to treat my patients with responsibility and criteria, since my education was based on earnest and well-established scientific evidence based upon proper groundworks. 

There, I was exhaustively trained to handle orthodontic wires by intensely making wire bends during several hours. To my view, this is the only possible way we can train truly responsible orthodontists.

Undoubtedly, properly trained orthodontic professionals require extensive training in study models with standard edgewise brackets that force them to perform all types of wire bends. Thus, when treatment requires such bends, the professional will not have any difficulties, regardless of the type of bracket used.

Those who are trained to properly use standard edgewise brackets are able to use all types of brackets, regardless of prescription. Conversely, those who are not properly trained to make 1^st^, 2^nd^, and 3^rd^ order bends are not able to properly treat patients, regardless of the type of bracket used. 

I believe every dental school should offer solid scientific guidance and basic training so as to allow students to master the technique, totally control the wire and, as a result, properly use the desired orthodontic accessories. 

I also believe that students should undergo in-lab training exclusively with standard edgewise brackets. Additionally, during training, the clinician must treat a few cases by means of the straight wire technique, but following the principles of biomechanics, regardless of the bracket. Thus, I believe students will be able to understand and have a critical eye towards the technique.

We need to praise the orthodontic technological development of wires and brackets, as they may bring major benefits to treatment. What we should reject; however, is having untrained students who believe that biomechanics is no longer necessary as brackets would solve everything.

I believe undergraduate and postgraduate courses should be based upon the essentials of biomechanics, diagnosis and planning. They should aim at well-established goals and have solidly trained students capable of completely handling orthodontic wire and appliance, as it is done at UFRJ.

Wrong diagnosis and planning with the use of the best appliances but without proper training yields disastrous results.^2^

## Based on your experience, do you notice any difference in the biological response of periodontally compromised teeth? Gerson Luiz Ulema Ribeiro

## What is the limit of treatment of periodontally compromised patients? Luiz Gonzaga Gandini Jr.

At first, all periodontally compromised cases may and must be treated, provided that periodontal disease is controlled. Success of orthodontic therapy relies on bone quality, not quantity.^3^

The basic rule for orthodontic movement in patients with little periodontal attachment is having healthy supporting tissues. Should there be no periodontal disease, we are dealing with teeth with less bone support, only. Therefore, the biological response will be the same, provided that the biomechanical system is adapted to the local features of these teeth.

Biological response does not depend on the amount of supporting tissue, but on the quality of these tissues^3^ and the biomechanical system applied Due to anatomical variations in alveolar supporting structures, orthodontic forces equal in magnitude yield great differences in the distribution of tension and pressure on tissues of different individuals.^4^

The amount and method of tooth movement resulting from the application of a system of forces relies not only on the magnitude, direction and characteristics of this system, but also on the points of force application in relation to the tooth as a whole.^5^

The orthodontist must be able to control the magnitude of force and the quality of the system applied to the tooth. Conversely, the speed and method of tooth movement are determined by biological response.^6^

Despite similar system of forces, the amount of tooth movement varies significantly from patient to patient and in the same patient.^7^^,^ 8 ^,^ 9 This may be due to differences in quality of the biomechanical systems applied; however, it also depends on the local variability of cell response.

In cases of little bone attachment, force is concentrated within a reduced periodontal area and, for this reason, may be over applied to normal periodontium.^10^^,^ 11 Should force be inadequately applied, there will be more hyalinization zones, less movement and more indirect resorption, which is a disaster for the already reduced periodontium.

Root control is hindered in teeth with little bone attachment, since the center of resistance is dislocated towards the apex. Thus, proper treatment of these patients requires detailed knowledge of biomechanics.

These cases cannot be treated by means of the straight wire technique because all teeth, whether periodontally compromised or not, would be subjected to equal biomechanical forces.

Orthodontic movement must be clearly avoided not only in cases of uncontrolled infection or inflammation, but also in teeth with little bone attachment without retention and stability at their new position.^12^

Periodontally compromised teeth should be treated with great care. In other words, with light forces and consistent biomechanical system applied to teeth with little bone attachment, thereby avoiding major movements and excessive proclination.^12^

When the patient presents periodontal health and hygiene during the active phase of treatment, there will be insignificant or no problems regarding bone support.^13^ However, in the absence of oral hygiene and presence of inflammation, there is a high risk of clinical attachment loss.^14^

On the other hand, many cases of bone loss do not yield good outcomes simply because the system of forces used was unsuitable for the case. In other words, improper forces were applied.

Therefore, good results are achieved when orthodontic movements are performed with light forces applied as near as possible to the center of resistance of teeth.^15^

## What are the underlying anchorage means and principles applied to orthodontic treatment of periodontally compromised patients? Gerson Luiz Ulema Ribeiro

Whenever the orthodontist is planning orthodontic treatment that includes application of a system of forces to a given movement, he must be fully aware of the need for anchorage in order to yield the desired outcomes.

Periodontally compromised patients, whose malocclusion is severed by major tooth migration, especially of incisor teeth, require the use of segmented arch for specific biomechanics. Major movements must be avoided, for this reason, final treatment outcomes do not consist in achieving perfect occlusion, but in improving function and yielding acceptable esthetic results, thereby controlling patient's periodontal disease.^10^

In general, premolars and canines present periodontium with the best conditions. Based on this hypothesis, I preferably use premolars, canines and, whenever possible, molars as anchorage. The ideal would be not to include these teeth in alignment and leveling or use passive archwire, since minor movements may interfere in periodontal proprioception, thereby changing occlusal sensation and decreasing anchorage efficiency.^10^

**Figure 3:**
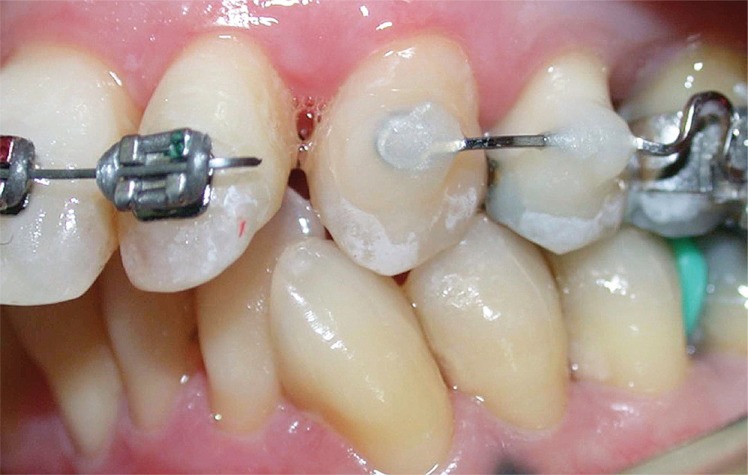
Anchorage with wire bonded directly to teeth.

**Figure 4:**
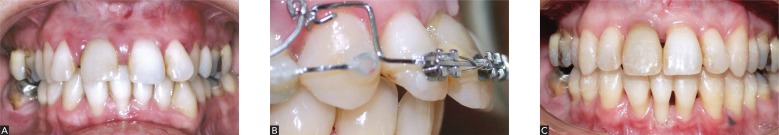
A) Clinical condition before treatment. B) Anchorage with wire bonded directly to teeth during incisors intrusion and retrusion by means of cantilevers. C) Final treatment outcomes

Thus, to achieve greater anchorage efficiency, 0.019 x 0.025-in steel wire is directly bonded to teeth without brackets and with transpalatal or lingual arch (Fig 3). This system uses occlusion as anchorage and allows us to perform movement of intrusion and/or retraction necessary for incisors (Fig 4).

**Figure 5:**
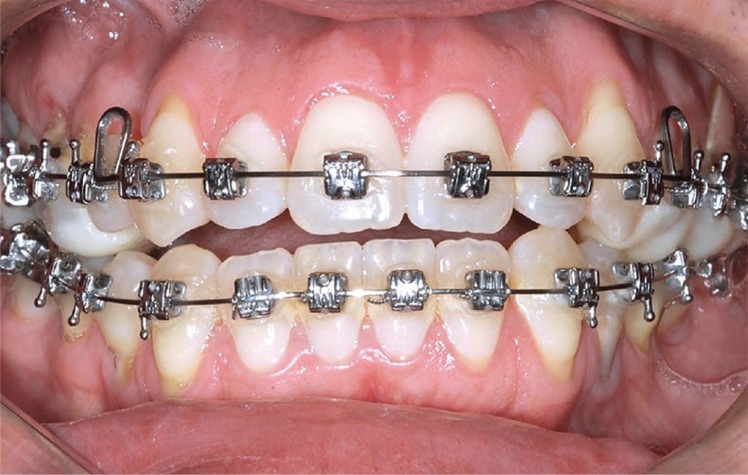
Triad resin used as anchorage for posterior teeth.

Occlusion may also be successfully used as anchorage by connecting all posterior teeth with Triad VLS resin (Dentsply, York, Pennsylvania, USA). The resin is inserted in the occlusal surface of teeth, and the patient is then required to occlude so as to interlock it. Subsequently, light is applied for curing. However, one must be careful not to exaggerate in the amount of resin and hinder hygiene. Triad Gel resin (Dentsply, York, Pennsylvania, USA) may also be used, as it favors hygiene. This splint maximizes the use of occlusal forces in anchorage, in addition to including teeth that were out of occlusion (Fig 5). Should it be necessary, leveling is conducted at treatment completion. 

Furthermore, should it be the case, Temporary Anchorage Devices (TADs) may be used directly or indirectly for anchorage of posterior teeth.

## Despite dental technical advance, is extraction still recommended for orthodontic purposes? Gerson Luiz Ulema Ribeiro

To my view, despite all technical advances, the clinician must be careful with the amount of tooth movement performed. It is limited in the posterior and anterior region as well as in the transverse dimension.

With the advent of TADs, many professionals are encouraged to perform major distal movements of second and first molars for later retraction of anterior teeth. Distal movement of molars is highly recommended in cases of dental discrepancy or molar correction that require movement not greater than 4 mm. Nevertheless, several cases require greater distalization. For instance, retreatment with previously extracted premolars.^16^^,^ 17

Distal tooth movement planning must consider the following factors:^18^

» Necessary space - with regard to efficiency of results, should each quadrant require more than 3 mm to achieve treatment goals, premolar extraction is preferable.

» Hard tissue conditions - there must be enough space for the posterior region. Extraction of second or third molars may be required to ensure proper space.

» Soft tissue conditions - there must be an acceptable amount of attached gingiva after distalization, particularly in the distal-vestibular region of lower second molar.

Orthodontic movement must respect the limits of bone bases. For the upper teeth, the tuberosity; and for lower teeth, the anterior edge of the ramus.^19^ Some cases will always require extractions to compensate for teeth crowding, incisor protrusion, which affects facial esthetics; and maxillomandibular discrepancy. It is probable that treatment without extraction and with expansion of dental arches has been taken to extremes. Once more, stability problems may be rendered important.^10^ We might begin to see relapses of these major movements in the medium and long term, which may hinder treatment outcomes. Likewise, rapid or slow dentoalveolar expansion imposes limits that must be respected; otherwise, relapses or periodontal problems may occur.

I believe that cases with mild discrepancy in which the use of TADs may be associated with expansion of the arch are well solved without extractions. As for cases of bimaxillary protrusion, I consider extraction to be necessary, particularly with the aid of TADs so as to prevent anchorage loss and, as a result, achieve maximum anterior retraction. 

In cases of dental arch asymmetry, asymmetric extractions ease treatment outcomes as they allow the use of symmetrical mechanics and eliminate the difficulties posed by asymmetric mechanics.^20^

Nevertheless, the role of extractions remains unclear, since there is not enough good scientific evidence to put an end to the matter.

## In which situations do you believe the use of straight archwires is less indicated than segmented mechanics? Luiz Gonzaga Gandini Jr.

Straight archwires must not be used in periodontal cases with severe malocclusion, given that they require specific mechanical systems.

In these cases, the four upper and/or lower incisors often undergo significant extrusion and buccal inclination; whereas in other cases, only one or two incisors are damaged and displaced differently from other teeth. Based on this hypothesis, there is a need for even more special and individualized care. It is of paramount importance that "sicker" teeth be treated with special care, with specific mechanical systems producing force and movement consistent with the amount of bone attachment. However, should the clinician not have enough knowledge of biomechanics to treat these cases or should straight wire be used alone, all teeth will be treated similarly and subjected to unwanted movement and force that hinder treatment as a result of greater bone loss.

**Figure 6:**
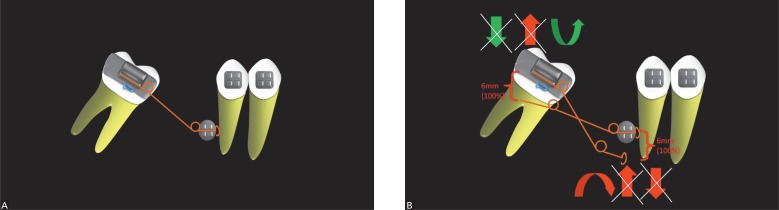
A) Uprighting spring adapted to the molar tube and the TAD canal. B) Uprighting spring activated in VI geometry. Note that the spring must be equally activated on both sides to eliminate forces.

**Figure 7:**
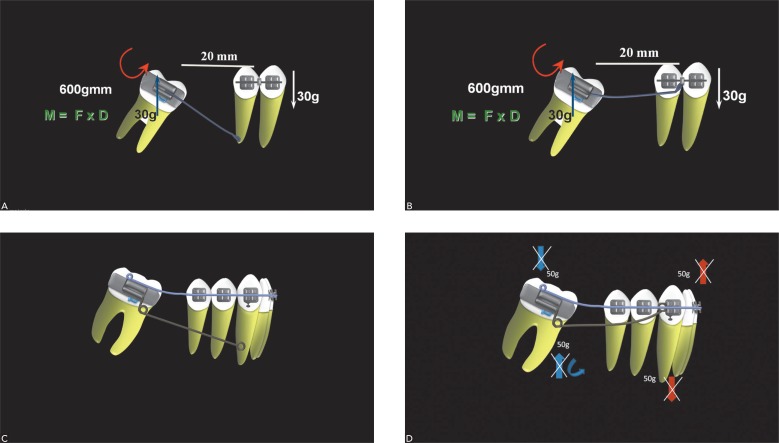
A, B) Cantilever used without further care regarding potential side effects. C, D) Care that should be taken to prevent molar extrusion. The end of the arch was used as cantilever tied to the molar tube.

In cases of molar uprighting, the use of straight wire, whether with superelastic wires or any type of loops, will result in molar extrusion which most of times is disastrous. Molars may be uprighted by activated springs in Burstone VI geometry that allows uprighting without extrusive forces. When the region of premolars is well anchored and as the system is deactivated with molar uprighting, intrusive force is incorporated into the molar, thereby rendering the system even more efficient (Fig 6).^21^ Cantilevers may also be used, provided that the extrusive effect they produce is controlled (Fig 7).

Similarly, straight wire should not be used in cases of curve of Spee in which incisor proclination is unwanted. Should straight wire be used with any type of bracket, the arch will follow the curve of Spee. We all know that the shortest distance between two points is a line. Therefore, the wire following the curve of Spee will be clearly longer.

For this reason, leveling the curve of Spee requires space. Should there not be enough space, teeth will undergo distal movement, in the transversal direction, or anterior movement with incisors proclination.

Many readers might ask themselves why I am discussing such a basic issue. I will explain: I have seen some "bracket-seller" professors suggesting that all orthodontic procedures are straightforward and, even though they know that the use of straight wire will result in incisor proclination, they teach their students the following: "Pay attention: to prevent incisor proclination, you must bend the end of the wire on the distal surface of molars."

However, if in front of molars the arch follows the curve of Spee, the latter is corrected with incisors proclination, not intrusion. Should the clinician opt to use straight wire with reverse curve of Spee, treatment will yield even more disastrous outcomes. Having to call attention to such a basic orthodontic issue is complete nonsense.

If orthodontic treatment goals include movement of teeth, and biomechanics is the branch of physics studying the effects of force, why is this field of study little emphasized in undergraduate orthodontic programs? Maurício Tatsuei Sakima

To my view, we should not treat this matter generically, since some undergraduate programs attach great importance to biomechanics, teaching it thoroughly and competently. 

However, with regard to undergraduate courses which do not cover this subject or cover it superficially and without scientific basis, I attribute this fact to irresponsible, dishonest and unskilled professors who teach their students that, since angulation, torque, in set and off set are incorporated to brackets, there is no need in having in-depth knowledge about biomechanics. According to these professors, everything can be solved simply and quickly. 

The marketing provided by these "professors" as well as by bracket industries has led to a real brainwashing. This advertising strategy has proved a big success, since students and clinicians long for treatment easiness attributed to appliances that supposedly allow treatment to be performed without in-depth training or knowledge.

Those students and clinicians only acknowledge they were deceived when they face complicated cases and are not able to solve them.

## What are the advantages of Burstone intrusion arch to treatment of periodontally compromised cases? Maurício Tatsuei Sakima

The major advantage of Burstone intrusion arch to treatment of periodontally compromised patients is that, when properly used as a device of the segmented arch technique, it allows true intrusion with mild and measurable forces (determined system of forces) and total root control without dental proclination, which would be a complete disaster for the periodontium.

To this end, the points of force application on each side must be well determined towards the center of resistance of the active member; in other words, towards the set of teeth to be intruded which must be joined as a single tooth with multiple roots. 

Oftentimes, to achieve success in these periodontal cases, force must be as little as possible (5 to 10 g per tooth)^7^ due to little periodontal attachment,^7^ given that the action of force will concentrate within a small periodontal area. Force considered as acceptable for teeth with healthy periodontium is a complete disaster for compromised ones. We should use systems that establish balance between moment and force with as little force as possible.

Differently from Ricketts base arch, Burstone intrusion arch is inserted into the bracket slot (indeterminate system of forces). As a result, force is applied ahead of the center of resistance of teeth. To control the tendency towards dental proclination, torque should be applied buccaly to the root. However, this is totally contraindicated for periodontally compromised patients, in which case it is difficult to determine necessary torque. 

Should intrusion planning include the use of straight wire, mechanics will be even more uncontrolled with excessively high forces and lack of root control, thereby totally hindering treatment.

Therefore, determined system of forces is undoubtedly more recommended due to offering perfectly controlled moment/force.

## Based on the biological reactions of orthodontic movement, what is the minimum visit schedule you recommend for periodontally compromised patients? Carla D'Agostini Derech

Since occlusal problems, treatment goals, local factors and periodontal damage vary considerably, the need for special care may require more accurate control of cases undergoing treatment. 

Furthermore, depending on the biomechanics used, there might be a need for monitoring the patient with frequency so as to asses the development of the system used. This does not mean the clinician should reactivate the system, but monitor the development of programmed movements instead.

Generally speaking, in routine cases, I usually see my patients every four weeks or 45 days. I have used heat-activated wires releasing lighter forces during longer periods of time.

Whenever we apply a given system of forces, we cannot avoid areas of hyalinization due to the irregularities found in periodontal space. In other words, force tends to be more appropriate in larger periodontal spaces, whereas it tends to be excessive and cause hyalinization in limited periodontal spaces.^6^

Thus, it is up to us to choose a biomechanical system that produces more appropriate and controlled movements, thereby minimizing hyalinization zones and yielding better outcomes within a shorter period of time.

**Figure 8:**
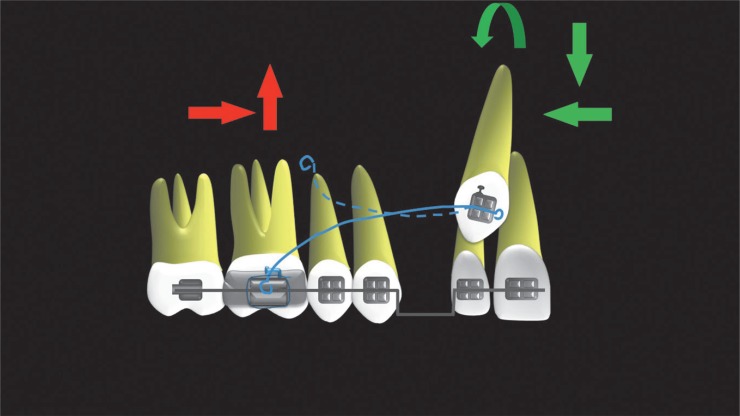
Cantilever used for root distalization, extrusion and correction.

**Figure 9:**
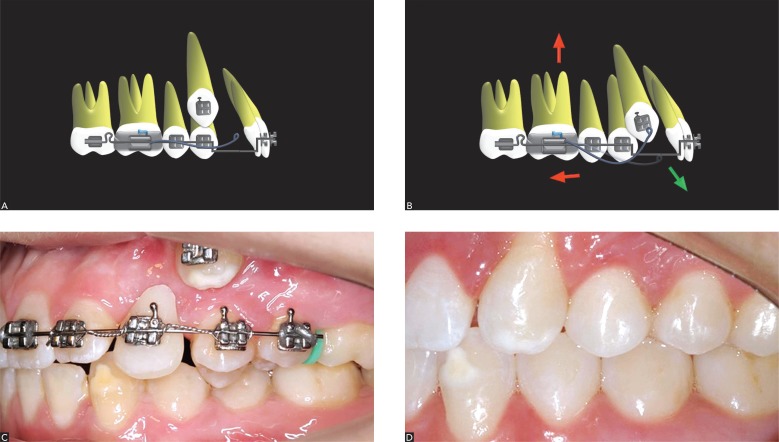
A, B) Cantilever used for canine extrusion and mesialization. C, D) Clinical case using cantilever for canine extrusion and mesialization.

The literature does not reach a consensus regarding the minimum force required for tooth movement to start nor the ideal force.^4^ The main point here is to use the best biomechanical system that produces appropriate force in the appropriate direction and points of application (Figs 8 and 9).

## With regard to orthodontic diagnosis of periodontally compromised adult patients, what is your current protocol on the use of imaging exams? Carla D'Agostini Derech

In fact, I do not have a definite protocol. Clinical exams vary depending on the severity of the case.

I routinely ask for lateral cephalometric radiograph, panoramic radiograph, as well as periapical and posterior interproximal survey. However, should clinical examination render necessary, I might ask for complementary tomographic examination in compromised areas. Should the case be generalized, I ask for complete tomographic examination so as to achieve a more accurate diagnosis.

I have received a massive amount of advertisements for orthodontic courses emphasizing technique as their major advantage in comparison to others. Additionally, they also advertise teaching the major bracket prescriptions and state-of-the-art appliances. Biology of Tooth Movement and Orthodontic Biomechanics are rarely included in these programs. What is your opinion about that? José Nelson Mucha

I believe this question has been answered in the aforementioned responses. However, I would like to emphasize that the biggest problem of courses advertising a certain type of bracket as the most modern and capable of yielding the best results is that the student is deceived and poorly trained.

The orthodontist must be the best. In other words, he must be well trained not only to apply biomechanics, but also to properly use the orthodontic accessories required by the case.

We need to take advantage of the technological development of which major objective is to provide patients with benefits. All bracket prescriptions, whether standard edgewise or pre-programmed, have their limitations because all cases require individualization, as we are dealing with human beings.

Should the type of appliance be emphasized, treatment is stereotyped and patient's individuality in terms of malocclusion and treatment goals is, therefore, forgotten. 

**Figure 10:**
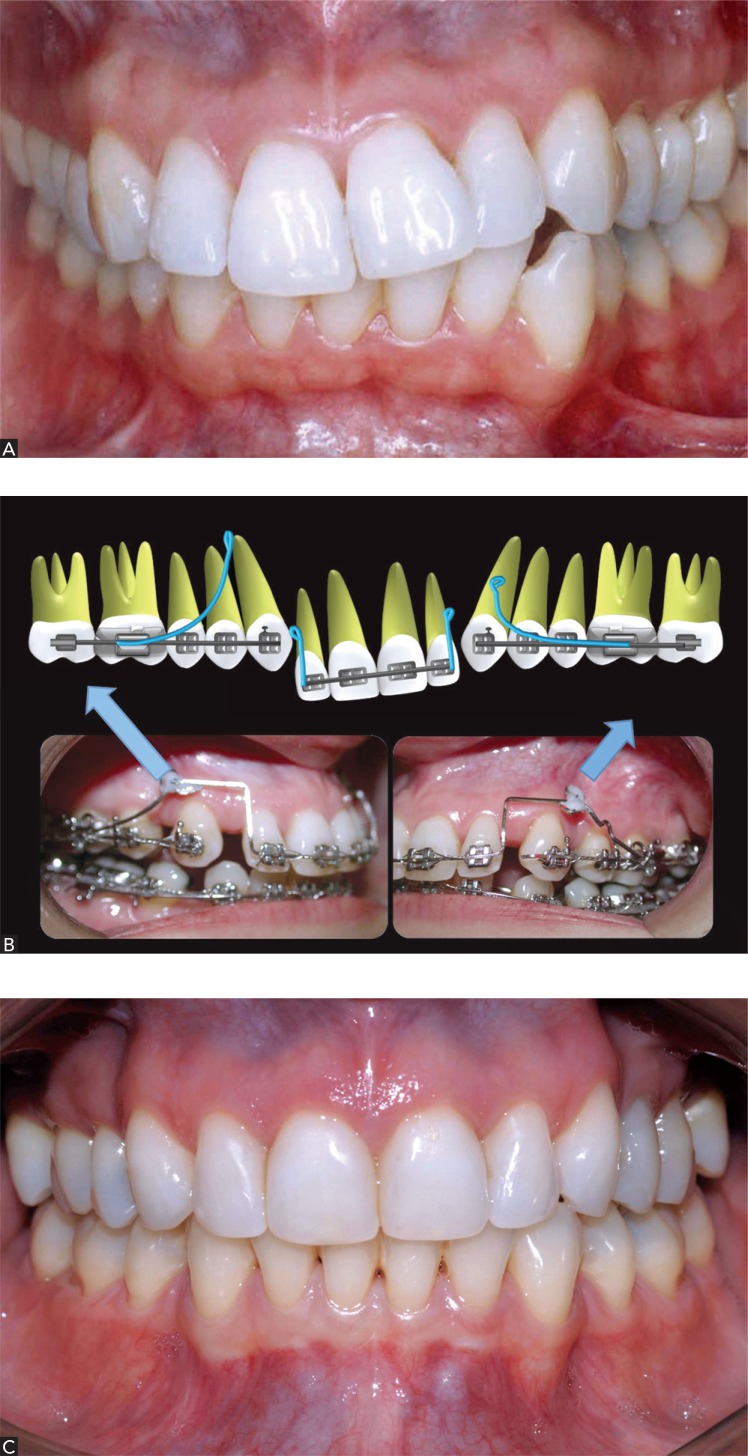
A) Clinical case in need of correction of the occlusal plane in the incisors area. B)Mechanics used for incisors intrusion and retrusion with different forces applied to correct the occlusal plane. C) Finished case.

**Figure 11:**
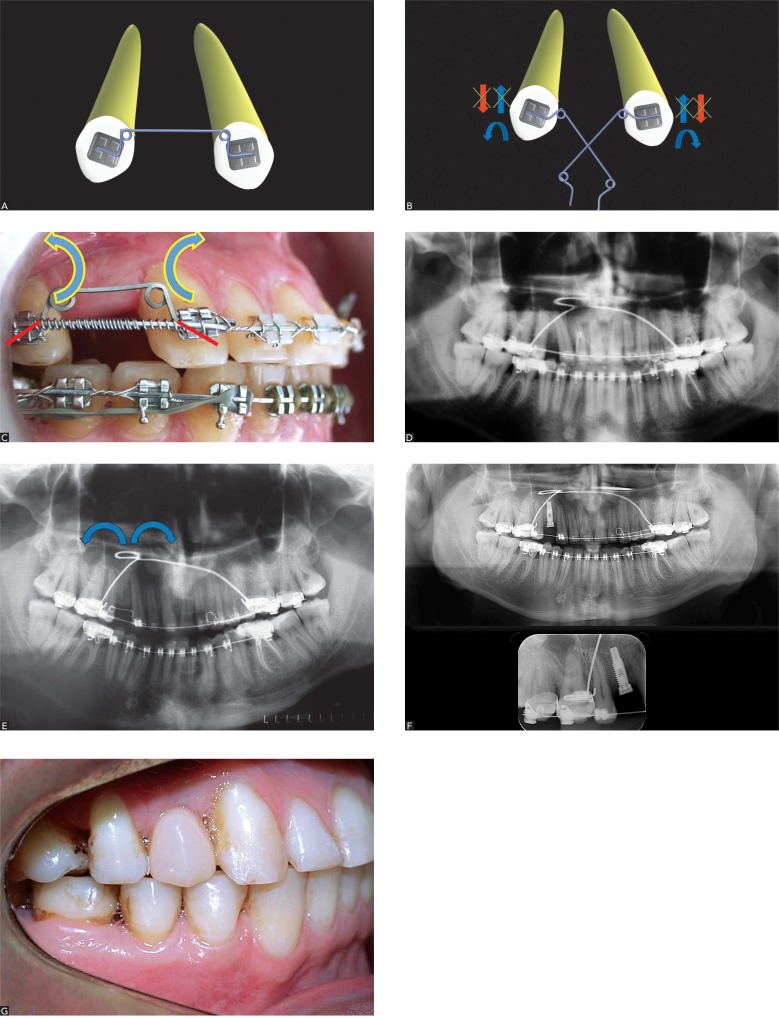
A, B) Mechanics used to split teeth root for implant placement. The procedure was carried out with spring activated in VI geometry. C-G) Clinical case showing mechanics performance.

A proper system of forces requires us to consider not only the effects produced by the appliance, but also the local factors involved, namely: different tooth shapes, varied bone support, root length and anatomical shape, and biological response^21^ (Figs 10 and 11).

Therefore, each case must be individually assessed so as to ensure that proper and most convenient biomechanics is applied, whether with segmented arch or straight wire.

During one of my courses on biomechanics, particularly aimed at specialists, I asked my students to manufacture a transpalatal arch for biomechanical training. What a surprise when one of my students raised his hand and humbly, but embarrassedly, reported not being able to do so, since he had not been trained to make bends and had never made a single one before. No words can describe such nonsense. To my view, that is shameful and dishonest.

Fortunately, by the end of the course, he was one of my best students who evinced intellectual ability and manual dexterity, both of which would have been lost if he had not aimed at broadening his knowledge.

Unfortunately, some students only realize they have been deceived when they face a severe problem they cause themselves or when they are incapable of solving more complex cases.

**Figure 12:**
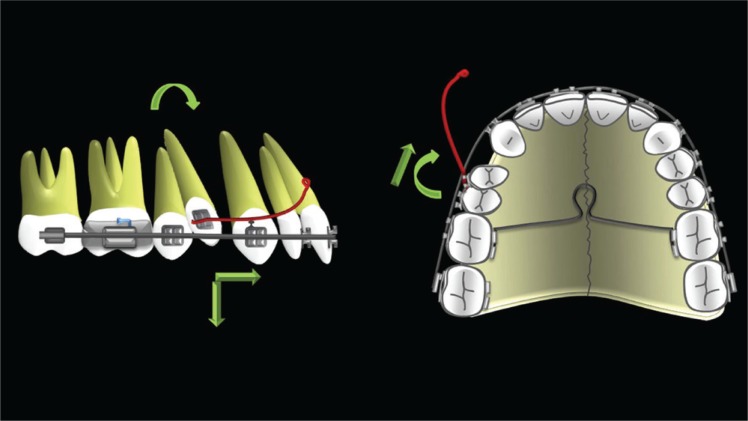
Cantilever used for root extrusion, mesialization and correction as well second premolar rotation.

Students or clinicians may use whatever bracket they are more familiarized with; however, the most important is knowing how to apply the biomechanics required for a specific case or tooth by means of using a proper system of forces (Fig 12).

Brazilian Orthodontics poses some doubts as to the number of courses and minimum credit hours (quality). It differs from the European Union where orthodontic courses have a minimum of 4,800 credit hours within 3 years, the World Federation of Orthodontics with a minimum of 3,700 hours and the United States where postgraduate programs have a minimum of 3,500 to 4,800 credit hours. How can we build a better future for Brazilian Orthodontics in terms of quantity and quality? José Nelson Mucha

The Brazilian Dental Association has fought tirelessly for quality of orthodontic postgraduate programs. Several meetings and discussions have been held with a view to reaching a consensus on how to improve and control those courses. They came to the conclusion that orthodontic courses should have a minimum of 2,000 credit hours, a reasonable number for Brazilian reality, which was already practiced in some good-quality programs. 

If that is the rule, all orthodontic professionals should strictly comply with it and provide students with proper in-lab training comprising a higher number of clinical cases and in-depth scientific evidence.

Another advantage brought by 2,000-hour courses is hindering the opening of programs of which professors and coordinators travel throughout Brazil irresponsibly offering their low-quality courses.

The quest for financial power is greater than the search for quality for which dedication, time and professional skills are necessary. Financial power significantly influences educational institutions that should also be concerned with quality. 

I remember that during one of the meetings held by the Brazilian Dental Association with representatives of the Brazilian Ministry of Education (MEC) and the Federal Council of Dentistry (CFO), we discussed about the need for agreeing on a minimum of 2,000 credit hours. The representatives were reluctant to understand and accept it, as if 2,000 hours were too much.

We had to convince them that those hours were strictly necessary. If we have difficulty in convincing our legal representatives that we need to adapt our programs to achieve higher quality, imagine "Dentistry businessmen".

However, despite the aforementioned difficulties, CFO agreed and issued a decree to regulate the minimum of 2,000 credit hours. Nevertheless, financial pressure remained and a new meeting has been recently called by CFO with a view to reopen discussion on the topic. And the prospect is not good.

To my view, poor management of Brazilian Dentistry begins in undergraduate programs. Since MEC allows the indiscriminate opening of undergraduate courses, it should impose qualification examination as an obligation for all graduates as a requirement to practise Dentistry, following the example of the Order of Attorneys of Brazil (OAB) and American Schools of Dentistry.

As for postgraduate programs, particularly in Orthodontics, students should also sit for a qualification examination, following the example of Brazilian Medicine and American Orthodontics in which the institutions representatives of specialities manage each domain.

That is the only solution to the serious problem faced by Brazilian Orthodontics. And that should be the major challenge of the Brazilian Dental Association.

Should the minimum amount of credit hours not remain as a result of financial and political pressure, students will be able to choose whatever course they wish, including those with reduced credit hours and hypothetically easy content. Nevertheless, should a qualification examination be imposed as an obligation for graduate and postgraduate students, potential applicants will take a fresh a look at their choice.

Thus, in response to your question, all we need is quality. Should there be strict and appropriate rules, without political or financial pressure, that not only exemplarily punish those who do not comply with their obligations, but are also combined with a qualification examination taken by the end of the course, a large amount of courses would certainly cease to exist.
